# Supplementary material for: Myeloid Fmr1 deficiency in mice results in reduced serum cholesterol and altered bile pathway gene expression
Source: PLoS One. 2026 Jan 12;21(1):e0340222. doi: 10.1371/journal.pone.0340222 (PMC12795373; doi:10.1371/journal.pone.0340222)
Supplement: S1 Table — (PDF) [file pone.0340222.s001.pdf]

Supporting information

S1 Table: Primers for mRNA analysis.

	Forward	Reverse
Fmr1	CCGAACAGATAATCGTCCACG	ACGCTGTCTGGCTTTTCCTTC
Cyp7a1	GAGAAGGCAAACGGGTGAAC	GGATTGGCACCAAATTGCAGA
Cyp7b1	CCCTCTTTCCTCCACTCATA	GAACCGATCGAACCTAAATTCCT
Cyp8b1	GCCTTCAAGTATGATCGGTTCCT	GATCTTCTTGCCCGACTTGTAGA
Cyp27a1	CTATGTGCTGCACTTGCCC	GGGCACTAGCCAGATTCACA
Ntcp	TTGCGCCATAGGGATCTTCC	ATCATGCCTGCCTTGAGGAC
Slco1a1	CTCCCATAATGCCCTTGGGT	GGGCCAACAATCTTCCCCAT
Slco1b2	GCACTGCGATGGATTCAGGA	CTCCACCTAGTGCCTTGCAT
Bsep	AGCAGGCTCAGCTGCATGAC	AATGGCCCGAGCAATAGCAA
Abcg5	CCTGAACATTCCAATCCCTTT	ATTCCTTGAAGGCACATTCC
Abcg8	AACACAAGCACCCACACAGT	CCGGAAGTCATTGGAAATCT
Cyp2b10	GACAGTTGCTGTCGTTGAGC	GGTCTTCCAACGTTCCCCAT
Mrp2	CAAATCCAATTCTCTACCTATGCAC	GCCTGCAGTGTTGGATCA
Sult2a8	ATGAGCGCACACCATGGATA	ATGAGACGTGGACCCTCCTT
Tgr5	TCCTGTCAGTCTTGGCCTATGA	GGTGCTGCCCAATGAGATG
