# Supplementary material for: Myeloid Fmr1 deficiency in mice results in reduced serum cholesterol and altered bile pathway gene expression
Source: PLoS One. 2026 Jan 12;21(1):e0340222. doi: 10.1371/journal.pone.0340222 (PMC12795373; doi:10.1371/journal.pone.0340222)
Supplement: S1 File — (PDF) [file pone.0340222.s002.pdf]

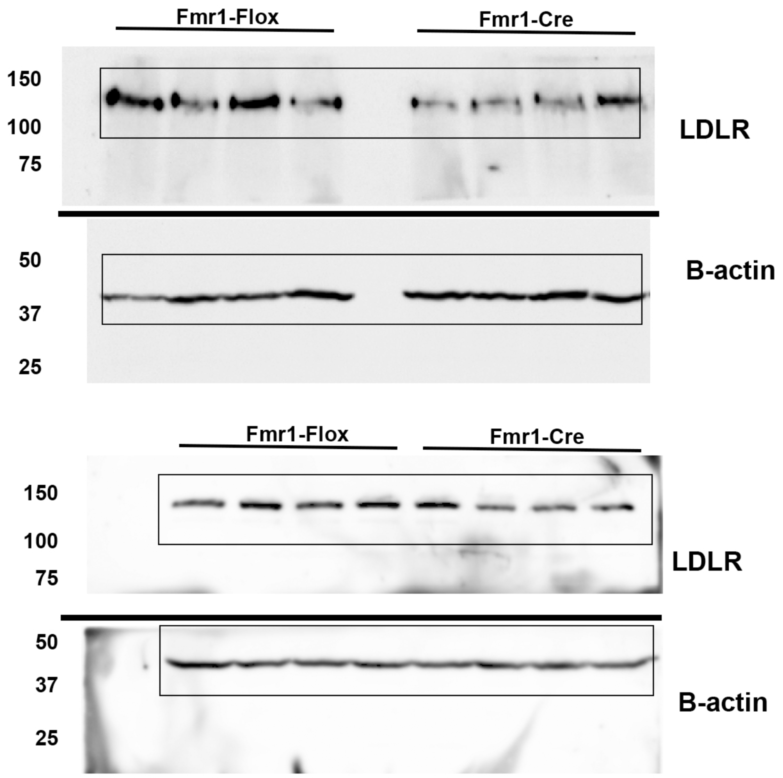

Western blot images for Fig 1: Box marks lanes used. Top panel depicts chemiluminescent Western blot for Fig 1C, bottom panel depicts chemiluminescent Western blot for Fig 1E. Images captured on Bio-Rad ChemiDoc Imaging System.

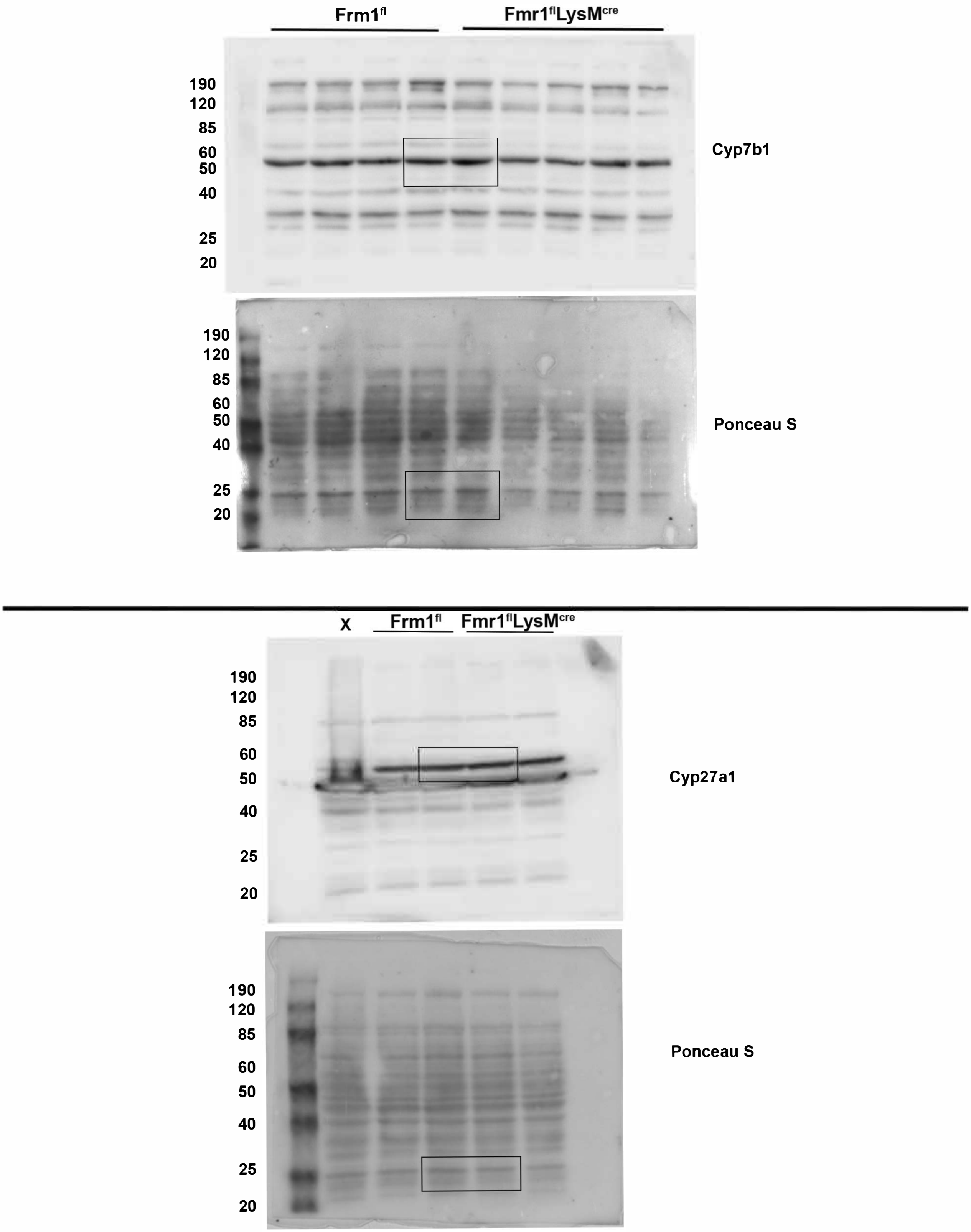

Western blot images For Fig 4: Boxed lanes are used in Fig 4A and 4C, respectively. Cyp7b1 and Cyp27a1 detected by chemiluminescent Western blot. Images captured on Bio-Rad ChemiDoc Imaging System.

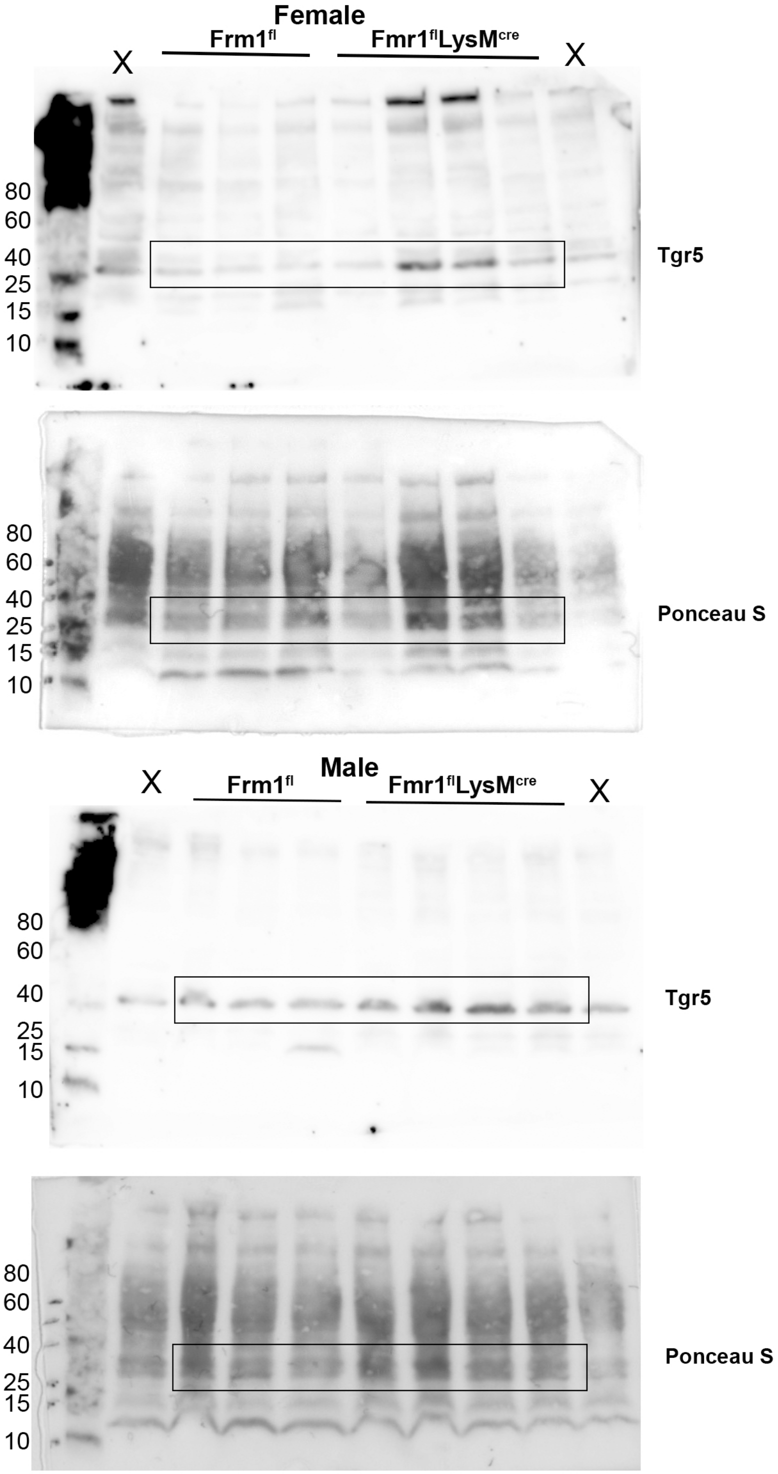

Western blot images For Fig 7B: Boxed lanes are used in Fig 7B. Tgr5 detected by chemiluminescent Western blot. Images captured on Bio-Rad ChemiDoc Imaging System.

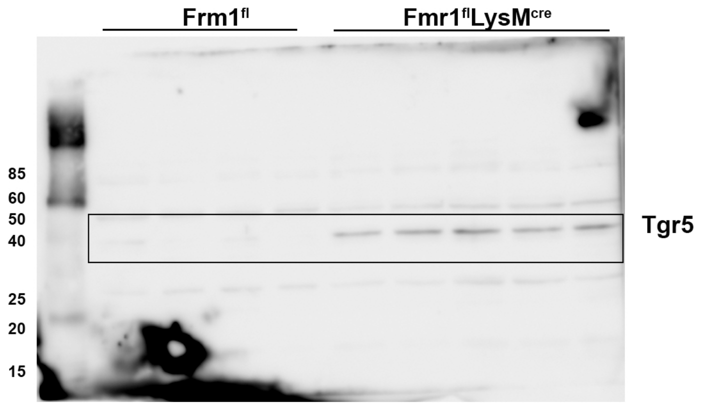

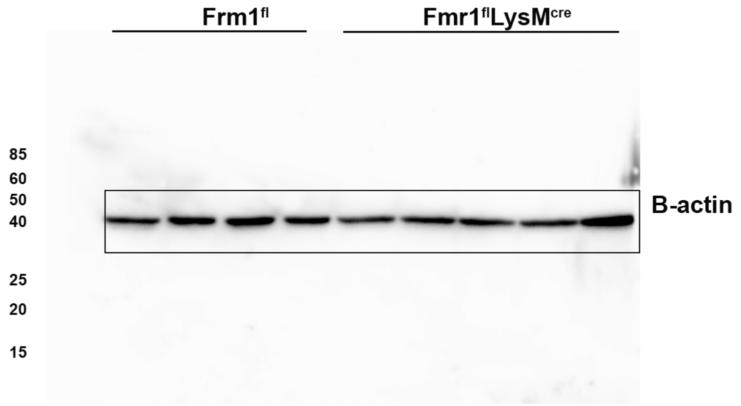

Western blot images for Fig 7F: Boxed lanes are used in Fig 7F. Tgr5 and B-actin detected with chemiluminescence Western blot. Images captured on Bio-Rad ChemiDoc Imaging System.
