# Supplementary material for: Myeloid Fmr1 deficiency in mice results in reduced serum cholesterol and altered bile pathway gene expression
Source: PLoS One. 2026 Jan 12;21(1):e0340222. doi: 10.1371/journal.pone.0340222 (PMC12795373; doi:10.1371/journal.pone.0340222)
Supplement: Attachment — Submitted filename: Response to Reviewers.pdf [file pone.0340222.s006.pdf]

Response to Reviewers' comments

PONE-D-25-43073
Myeloid Fmr1 deficiency in mice results in reduced serum cholesterol and altered bile pathway gene expression

We thank the Reviewers for their constructive comments and suggestions for improvements of the manuscript. Below is a point-by-point response to the comments with reference to additional experiments as appropriate.

Reviewer #1:

Materials and methods: Animals (and other sections of the manuscript):

1. *"total Fmr1-/- deficiency"* – please use "Fmr1-KO mice"

Fmr1-KO is now used for total Fmr1-/- throughout the manuscript.

2. Specify endpoints; in the following text, you are measuring the cholesterol, etc., in serum. When did you collect the blood: in vivo or ex vivo.

Blood collection for serum was performed immediately before euthanasia. This is now stated in the Methods section (line 117).

3. 77-78: *Fmr1-flox were crossed with LysMcre purchased from Jackson Lab to generate 79: myeloid specific Fmr1 deficient mice (Fmr1-cre)* – Please specify if you crossed and bred the mice in-house or if someone else did it.

Fmr1-fl were crossed with Fmr1-cre mice by our collaborator and bred in-house. This is now stated with citation in the Methods section (line 77-80).

4. *79: Mice of both sexes were used.* – Please change to: Male and female mice were utilized in our experiments.

This has been changed in Methods section (line 80-81).

5. 79-80: *Some mice were fed Western diet (TD.88137, Envigo) starting at 7 weeks of age for 6 weeks.*

- Please specify what it means: Western diet.
- Please specify the control diet.
- Did you randomize mice for the diet?
- "Some mice…" It is not scientific. Please rewrite the sentence.

Control and Western diets and randomization are described in Methods, line 81-83.

6. 84 change the title for: gene expression analysis using RT-qPCR.

The title has been changed (line 91).

7. 85: *Total RNA was isolated using TRIzol.* Please describe the method in brief.

The method is now described in brief (Methods, lines 92-96).

8. 88: Specify the cycle program for qPCR.

qPCR cycle program is now stated (Methods, line 99-100).

9. 89: "*ΔΔCt method.*" Add references

Reference has been added in References (line 489).

10. Supplementary table 1: Please, stay with one selected by you format for sequence description: constant or using trinucleotide format.

Primer format is now consistent (Supplementary Table 1).

11. 93: *protein concentration was obtained,* - Please specify the method.

Method of protein quantification is now stated (Methods, line 106).

12. *105-106: "Bile acid levels in"*- Please specify which bile acids were measured.

Total bile acids were measured (Methods, line 120).

13. 114-121: BMDMs isolation. Please see methods described in https://www.biorxiv.org/content/10.1101/2025.02.19.638515v1.full and adjust the BMDMs isolation accordingly.

Method description has been adjusted as appropriate (Methods, lines 129-136).

14. Statistic: Please use SEM instead of SD in the presented results.

Data are now presented as Mean +/- SEM and stated as such in Methods (line 147).

15. Figure 1B should be supplemented with mice weight measurement throughout the experiment time.

Unfortunately, weights were only measured at the end of the experimental protocol.

16. Fig 1C-D. It would be beneficial to assess the expression of LDLR in mice treated with a control diet.

We have added the Western blot analysis of mice fed normal chow (Fig 1C & 1D). There was no significant difference between the genotypes.

17. 140-141: "*suggesting that reduced PCSK9 function was not involved in the observed reduction in"*- To make that conclusion, it is recommended to evaluate the protein expression of PCSK9.

We have removed this statement from the manuscript.

18. All figures:
    Please specify the number of mice (n=?) used in the experiments.
    Please use colored graphs, as it will be easier to track between the figures.

Figure legends have been revised to include N. Graphs are now color-coded. We thank the reviewer for these helpful suggestions.

19. Why Fig1 and fig 2 present results only from male mice and not female? Where you follow the study using male and female mice.

The experiments with Fmr1-KO mice were performed only in male mice to confirm if the reported changes in bile acid synthesis pathway gene expression in male Fmr1-KO mice (Leboucher et al, Mol Metab. 2019 Mar:21:22-35; doi: 10.1016/j.molmet.2019.01.002) could be replicated in our lab. Once this pathway was confirmed as a potential mechanism for the observed reduction in serum cholesterol, the studies were then continued with both female and male mice using the $Fmr1^{fl}$ model with $LysM^{cre}$.

20. Fig3. Normal chow – is it the chow from the animal facility or control to the Western diet?

Normal chow is the standard diet in the animal facility.

21. Fig. 3. Please add human data or elaborate in text about cholesterol levels in males and females.

Unfortunately, we do not have access to data of humans in a study comparing effect of diets and sex on cholesterol levels. We have added a paragraph in the Discussion (lines 383-386) discussing factors involved in reduced cholesterol levels in females.

22. According to Human Protein Atlas and immune cell selection, FMR1 is highly expressed in neutrophils. https://www.proteinatlas.org/ENSG00000102081-FMR1/single+cell
Why did you decide to evaluate the BMDMs?

We thank the Reviewer for this comment. At present time, we have not found reports that support a role for neutrophil Fmr1 in lipid metabolism and this can be part of the next phase of our work. However, the comment did lead us to evaluate the expression of Tgr5 in peritoneal neutrophils. The peritoneal cavity cells were not activated by use of thioglycolate and instead isolated by washing only with PBS/0.5mM EDTA buffer. While we did find Tgr5 expressed in non-activated peritoneal cavity neutrophils (Ly6G+ cells), the number of neutrophils in half of the mice tested were extremely low to be able to make a definitive conclusion. Please see Figure 1 below:

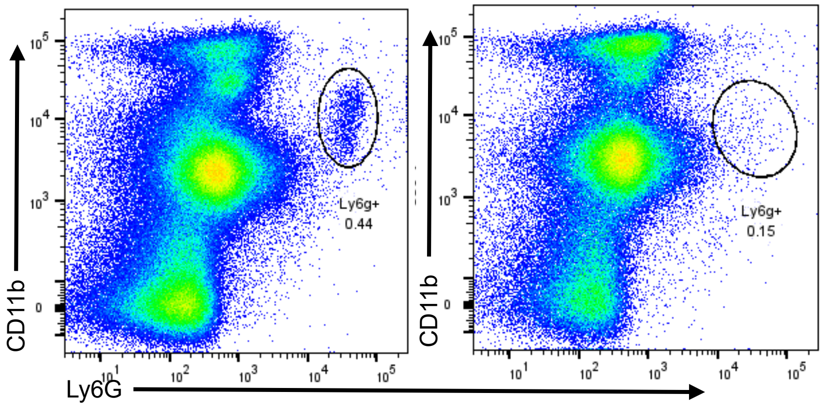

Figure 1: Representative FACS result of Ly6G+ neutrophils in peritoneal cavity cell isolation. Left panel depicts distinct cell cluster of CD11b$^-$Ly6G+ cells in 1 mouse whereas panel on the right depicts a lack of cell cluster in another. Cells were stained at the same experiment using a master antibody mix.

Experiments in the future will need to address these technical issues.

23. Fig.7A – please remove "##" from the figure.
    Fig.7B – Please, upload a higher resolution of western blot pictures.

The pound signs have been removed from the figure. We have uploaded Western blot photos with higher contrast setting.

24. The authors focus on BMDMs and cholesterol metabolism, which is also connected with different cancers – please comment on that in the discussion. Are "*Fmr1 and the metabolic pathways mediated by Tgr5"* implicated in cancer development and progression?

We thank the Reviewer for the comment that could potentially link Fmr1-Tgr5 interaction with different cancers. The consensus is somewhat more nuanced, but we have included a short discussion of the relevance (Discussion, lines 438-442).

Reviewer #2:
We thank the Reviewer for the positive comments and requests for clarification to further enhance the report.

1. You did a great experiment by overexpressing Sult2a8 and showing it does not change cholesterol on its own. This honestly makes the story more interesting because it means the reduced cholesterol is not just a simple result of bile acid detoxification. You should highlight this more in the discussion. It's a negative result that positively pushes the narrative forward, forcing you to look downstream (which led you to Tgr5).

We have added some text in the Discussion to further elaborate on the approach (lines 389-397).

2. Serum bile acids: Despite altered bile synthesis gene expression, no significant differences were observed in bile acid levels. This discrepancy is underexplored and weakens the connection between the gene expression data and physiology.

We appreciate this discrepancy and is the reason we continued to follow the pathway to its sequential steps. We agree that it is underexplored but will need significantly more time and additional expertise to unravel. This is planned for future studies in our lab.

3. **Functional assays missing**: No direct test of Tgr5 activity (e.g., downstream signaling, agonist/antagonist experiments). No metabolic/behavioral endpoints beyond cholesterol (e.g., glucose tolerance, liver histology, inflammatory markers).

We have performed qPCR to evaluate mRNA levels of IL-1β and IL-6 in livers of the mice (Fig 7C & 7D). The only difference was found in reduced IL-6 expression in male $Fmr1^{fl}$-$LysM^{cre}$ mice compared to male $Fmr1^{fl}$ mice. Thus, the pathways would seem complex with some potential sex differences. We performed an additional preliminary experiment to evaluate the effect of activating the Tgr5 signaling pathway on Fmr1 expression using the Tgr5 agonist INT-777 to treat BMDM from male mice and found that INT-777 significantly reduced Fmr1 mRNA expression in Fmr1-fl mice (see below). Although preliminary, combined with the manuscript data, it suggests that Fmr1 and Tgr5 regulate each other in a potential negative feedback pathway but this needs further confirmation.

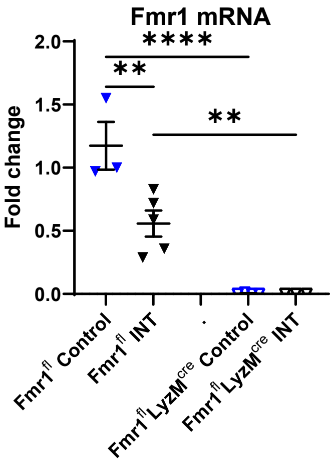

Figure 2: Effect of Tgr5 agonist INT-777 (INT) on Fmr1 mRNA expression in BMDM from male Fmr1^{fl} mice and Fmr1^{fl}LysM^{cre}mice. **P<0.01; ****P<0.0001

4. **Human relevance**: The discussion acknowledges differences between mice and humans, but the translational potential could be elaborated further (for example: whether bile acid/TGR5 pathways in humans are similarly regulated).

We have added this to the Discussion (lines 431-437) and state that the Tgr5 function is highly conserved between mice and humans. We thank the reviewer for this helpful suggestion.

5. **Mechanistic Speculation:** The discussion on how myeloid Fmr1 deficiency leads to increased hepatic Tgr5 expression is necessarily speculative. While this is fine for a discussion, explicitly framing it as a key question for future research would strengthen the manuscript. Is a soluble factor from Fmr1-deficient macrophages acting on hepatocytes in a paracrine manner?

We have added text in the Discussion (line 420-430) to expand on this. We thank the Reviewer for this suggestion to strengthen the manuscript.

6. **Quantifying the Myeloid Specific Deletion**: This is a minor point, but it would add strength. How efficient was the Fmr1 deletion in myeloid cells? Showing a qPCR or Western blot confirming a strong reduction of FMRP in the bone marrow derived macrophages (BMDMs) or in isolated liver macrophages (Kupffer cells) from the Fmr1cre mice would nicely validate your model.

We have added Fmr1 qPCR results with BMDM in Fig 3A to confirm the Fmr1 deficient phenotype.

7. **The Tgr5 Connection**: This is the most exciting part. The increased Tgr5 expression in the livers and BMDMs of Fmr1 cre mice is a fantastic clue.

- **Mechanism:** The discussion rightly says the mechanism is unclear. Could FMRP normally bind to and suppress Tgr5 mRNA? A simple experiment in the BMDMs could test this: does Tgr5 mRNA have a longer half-life in the Fmr1cre cells? This could be a future direction.

- **Function:** Is the increased Tgr5 functional? A future study could see if macrophages from Fmr1-cre mice produce more cAMP (a key downstream signal of Tgr5 activation) when exposed to bile acids.

We appreciate the Reviewer's assessment of the results and potential significance, of which summarizes our enthusiasm for future studies. As we show in the attached figure above, there does appear to be some negative regulatory loop that involves Fmr1 and Tgr5, at least in the BMDM cells tested. However, much is left to do to understand this pathway.
