# Supplementary material for: Myeloid Fmr1 deficiency in mice results in reduced serum cholesterol and altered bile pathway gene expression
Source: PLoS One. 2026 Jan 12;21(1):e0340222. doi: 10.1371/journal.pone.0340222 (PMC12795373; doi:10.1371/journal.pone.0340222)
Supplement: Attachment — Submitted filename: PONE-D-25-43073_reviewer.docx [file pone.0340222.s003.docx]

In the presented manuscript authors try to assess the role of total deficiency compared to myeloid cell-specific deficiency of the Fmr1 gene in modulating cholesterol levels in mice. The investigation focused on the context of Western diet, which has a detrimental effect on metabolism. The results show that myeloid-specific deficiency of Fmr1 recapitulates the lipid metabolism phenotype of total Fmr1 deficiency. The results also suggest that the altered lipid metabolic phenotype of Fmr1 deficiency clusters around the bile acid receptor Takeda G protein-coupled membrane receptor (Tgr5)

Even though the presented here study has an overall impact, it needs some adjustments to the text and additional experiments are needed.

Materials and methods: Animals (and other sections of the manuscript):

*“total Fmr1-/-* *deficiency”* – please use “Fmr1-KO mice”

Specify endpoints; in the following text, you are measuring the cholesterol, etc., in serum. When did you collect the blood: in vivo or ex vivo.

78-77: *Fmr1-flox were crossed with LysMcre purchased from Jackson Lab to generate*

*79: myeloid specific Fmr1 deficient mice (Fmr1-cre) –* Please specify if you crossed and bred the mice in-house or if someone else did it.

*Fmr1-cre –* Please, at least in the method section, use full mouse strain name: *Fmr1 fl/fl LysMcre.* It will help track the experiments.

*79: Mice of both sexes were used. –* Please change to: Male and female mice were utilized in our experiments.

79-80: *Some mice were fed Western diet (TD.88137, Envigo) starting at 7 weeks of age for 6 weeks.*

1. Please specify what it means: Western diet.
2. Please specify the control diet.
3. Did you randomize mice for the diet?
4. “Some mice…” It is not scientific. Please rewrite the sentence.

84 change the title for: gene expression analysis using RT-qPCR

85: *Total RNA was isolated using TRIzol.* Please describe the method in brief.

88: Specify the cycle program for qPCR.

89: “*ΔΔCt method.”* Add references

Supplementary table 1: Please, stay with one selected by you format for sequence description: constant or using trinucleotide format.

93: *protein concentration was obtained, -* Please specify the method.

*105-106: “Bile acid levels in”-* Please specify which bile acids were measured.

114-121: BMDMs isolation. Please see methods described in <https://www.biorxiv.org/content/10.1101/2025.02.19.638515v1.full> and adjust the BMDMs isolation accordingly.

Statistic: Please use SEM instead of SD in the presented results.

Figure 1B should be supplemented with mice weight measurement throughout the experiment time.

Fig 1C-D. It would be beneficial to assess the expression of LDLR in mice treated with a control diet.

140-141: “*suggesting that reduced PCSK9 function was not involved in the observed reduction in”-* To make that conclusion, it is recommended to evaluate the protein expression of PCSK9.

All figures:

Please specify the number of mice (n=?) used in the experiments.

Please use colored graphs, as it will be easier to track between the figures.

Why Fig1 and fig 2 present results only from male mice and not female? Where you follow the study using male and female mice.

Fig3. Normal chaw – is it the chaw from the animal facility or control to the Western diet?

Fig. 3. Please add human data or elaborate in text about cholesterol levels in males and females.

According to Human Protein Atlas and immune cell selection, FMR1 is highly expressed in neutrophils. <https://www.proteinatlas.org/ENSG00000102081-FMR1/single+cell>

Why did you decide to evaluate the BMDMs?

Fig.7A – please remove “##” from the figure.

Fig.7B – Please, upload a higher resolution of western blot pictures.

Fig.7C – it would be valuable to perform protein analysis of *Tgr5* in BMDMs from *Fmr1-cre Fmr1-flox* male and female.

The authors focus on BMDMs and cholesterol metabolism, which is also connected with different cancers – please comment on that in the discussion. Are “*Fmr1 and the metabolic pathways mediated by Tgr5”* implicated in cancer development and progression?
