# Supplementary material for: Myeloid Fmr1 deficiency in mice results in reduced serum cholesterol and altered bile pathway gene expression
Source: PLoS One. 2026 Jan 12;21(1):e0340222. doi: 10.1371/journal.pone.0340222 (PMC12795373; doi:10.1371/journal.pone.0340222)
Supplement: Attachment — Submitted filename: Comments for paper.docx [file pone.0340222.s004.docx]

**Overall Impression**

This is a well written and logically structured manuscript that presents a novel and fascinating investigation into the role of myeloid specific Fmr1 deficiency in cholesterol metabolism. The study effectively builds upon known phenotypes of total Fmr1 deficiency (reduced serum cholesterol) and successfully identifies a specific immune cell lineage as a key mediator. The most significant finding is the proposed novel link between Fmr1 deficiency and the bile acid receptor Tgr5, which opens a new avenue for therapeutic research. The work is technically sound, with appropriate controls and methodologies.

**Major Strengths:**

1. The focus on myeloid specific Fmr1 knockout to dissect the metabolic phenotype is innovative and addresses an important gap in the field regarding cell type specific effects.
2. The data robustly support the main conclusions. The replication of the hypocholesterolemic phenotype in the conditional knockout model is convincing. The comprehensive analysis of bile acid synthesis, transport, and detoxification genes provides a thorough mechanistic exploration.
3. The inclusion and analysis of both male and female mice throughout the study is a major strength.
4. The discovery of consistently elevated Tgr5 expression in Fmr1 deficient mice (both in liver and BMDMs) is a standout finding with significant potential implications for understanding the metabolic aspects of Fragile X Syndrome.

**Constructive Comments & Questions for the Authors**

Here are some thoughts and questions, framed to help strengthen the manuscript even further.

1. You did a great experiment by overexpressing Sult2a8 and showing it does not change cholesterol on its own. This honestly makes the story more interesting because it means the reduced cholesterol is not just a simple result of bile acid detoxification. You should highlight this more in the discussion. It's a negative result that positively pushes the narrative forward, forcing you to look downstream (which led you to Tgr5).

2. **Serum bile acids**: Despite altered bile synthesis gene expression, no significant differences were observed in bile acid levels. This discrepancy is underexplored and weakens the connection between the gene expression data and physiology.

3. **Functional assays missing**: No direct test of Tgr5 activity (e.g., downstream signaling, agonist/antagonist experiments). No metabolic/behavioral endpoints beyond cholesterol (e.g., glucose tolerance, liver histology, inflammatory markers).

4. **Human relevance**: The discussion acknowledges differences between mice and humans, but the translational potential could be elaborated further (for example: whether bile acid/TGR5 pathways in humans are similarly regulated).

5. **Mechanistic Speculation:** The discussion on how myeloid Fmr1 deficiency leads to increased hepatic Tgr5 expression is necessarily speculative. While this is fine for a discussion, explicitly framing it as a key question for future research would strengthen the manuscript. Is a soluble factor from Fmr1-deficient macrophages acting on hepatocytes in a paracrine manner?

6. **Quantifying the Myeloid Specific Deletion**: This is a minor point, but it would add strength. How efficient was the Fmr1 deletion in myeloid cells? Showing a qPCR or Western blot confirming a strong reduction of FMRP in the bone marrow derived macrophages (BMDMs) or in isolated liver macrophages (Kupffer cells) from the Fmr1cre mice would nicely validate your model.

7. **The Tgr5 Connection**: This is the most exciting part. The increased Tgr5 expression in the livers and BMDMs of Fmr1 cre mice is a fantastic clue.

- **Mechanism:** The discussion rightly says the mechanism is unclear. Could FMRP normally bind to and suppress Tgr5 mRNA? A simple experiment in the BMDMs could test this: does Tgr5 mRNA have a longer half-life in the Fmr1cre cells? This could be a future direction.
- **Function:** Is the increased Tgr5 functional? A future study could see if macrophages from Fmr1-cre mice produce more cAMP (a key downstream signal of Tgr5 activation) when exposed to bile acids.

**Summary for the Authors**

Authors have done excellent work here. Convincingly shown that:

1. Myeloid cells are key players in the low-cholesterol phenotype of Fmr1 deficiency.
2. This happens through altered bile acid pathway gene expression in a sex specific way.
3. The bile acid receptor Tgr5 is likely a major player in this process.

After minor revision manuscript is very close to being ready for publication. This is a solid contribution to the fields of metabolism, immunology, and Fragile X research.
